# Insomnia and hearing impairment among occupational noise exposed male workers

**DOI:** 10.1186/s40557-017-0195-7

**Published:** 2017-08-15

**Authors:** Hyeong-Min Lim, WonYang Kang, Won-Ju Park, Keun-Ho Jang, Ji-Sung Ann, Jai-Dong Moon

**Affiliations:** 1 Department of Occupational and Environmental Medicine, Chonnam National University Hwasun Hospital, 322 Seoyang-ro, Hwasun-gun, Jeollanam-do, 58128 Republic of Korea; 2 Department of Occupational and Environmental Medicine, Mokpo Christian Hospital, 303 Baengnyeon-daero, Mokpo-si, Jeollanam-do, Republic of Korea

**Keywords:** Hearing loss, Insomnia, Noise, Sleep

## Abstract

**Background:**

The aim of this study was to investigate the association between insomnia and hearing impairment among workers exposed to occupational noise.

**Methods:**

This study included 809 male workers exposed to occupational noise. The participants underwent audiometric testing, and their insomnia was examined based on the Insomnia Severity Index test. Hearing impairment was defined as hearing threshold >25 dB hearing level in the range of 1–4 kHz.

**Results:**

According to analysis of covariance, it was observed that pure tone audiometry thresholds at 1–2 kHz in the right ear and at 1 kHz in the left ear were significantly higher among workers with insomnia compared to those with no insomnia. Multiple logistic regression analysis of insomnia for hearing impairments was performed, which showed the odds ratio was 1.806 (95% confidence intervals: 1.022–3.188, *p* = 0.042) after adjustment for age, working period, noise level, snoring, use of protection devices, diabetes mellitus, hypertension, smoking, alcohol consumption, regular exercise, waist circumference, total cholesterol, triglyceride and high density lipoprotein cholesterol.

**Conclusion:**

Insomnia could be associated with hearing impairment in workers who are exposed to occupational noise. Additionally, insomnia may be associated with decreased hearing at low frequencies. Especially, more efforts are required to improve the quality of sleep for workers who are exposed to loud occupational noise. Further well- designed prospective studies are needed to clarify the relationship between insomnia and hearing impairment.

## Background

Noise-induced hearing loss (NIHL) is one of the most prevalent occupational disorders worldwide. It is estimated that about 16% of the cases of adult-onset hearing loss are due to induced by noise [1]. In 1991, Korean workers with NIHL accounted for 55.5% of the total workers with occupational diseases, and its incidence has increased continuously. According to a report in 2014 by the Korean Ministry of Employment and Labor, among workers with occupational disease, 95.3% were NIHL [2].

NIHL has deleterious effects on workers such as social disconnection, depression, and increased risks of accidents. Further, it raises social expenditure for treatment, compensation, and rehabilitation [3]. Various strategies have been adopted for preventing NIHL; for instance, periodic noise monitoring in workplace, use of hearing protection devices, etc. However, there is considerable discrepancy in the sensitivity for NIHL among individuals. Some individuals might experience hearing loss when exposed to short-term noise, whears others might tolerate intense loud noise without any hearing loss. This is because of the differences in individual’s susceptibility, and some studies have been conducted to understand the cause of these differences [4, 5].

Insomnia is a common problem in adults and is defined as a subjective feeling of having difficulty in initiating or maintaining sleep or having poor sleep quality [6]. It has been reported that prevalence of insomnia ranges from 10 to 30% in the western countries [7–9]. Being rarely reported in the eastern countries compared to the western countries, prevalence of insomnia was 15.3% in a Singaporean study, 21.4% in a Japanese study respectively [10, 11]. Additionally, in 2005, a Korean study revealed that 22.8% of the 5000 subjects aged between 20 and 69 years experienced insomnia [12]. Several studies showed that insomnia causes harmful effects and is associated with impairments of life quality, mental health, accident occurrence, and circulatory problems such as myocardial infarction and heart failure [13–17].

Only few studies have been conducted to examine the association between sleep and hearing loss. A study in Sweden indicated poorer sleep quality is associated with higher prevalence of hearing problems. However, this study focused on not only hearing loss but also considering subjective hearing complaints as hearing problems [18]. Another cross-sectional study in Israel showed association between hearing impairment and sleep quality; however, this study indicated influence of hearing impairment on sleep quality [19]. A study conducted on Korean workers exposed to aircraft noise revealed no significant correlation between sleep quality and NIHL [20]. As above, former studies have been conducted to examine the relationship between sleep quality and hearing loss. Also, these studies showed inconsistent results and did not exclusively focus on insomnia (that is, overall sleep disturbance was taken into account). Thus, association between sleep disturbances, including insomnia, and hearing loss has not been elucidated clearly.

We therefore conducted this study to examine the relationship between insomnia by using the Insomnia Severity Index (ISI) and hearing impairment objectively assessed by audiometric device among occupational noise exposed workers.

## Methods

### Study subjects

This study was conducted on 840 male workers, aged between 25 and 60 years, at a tire-manufacturing factory. They underwent specific health examinations from October 2014 to November 2014. We excluded 31 workers with a history of ear diseases or with abnormal lesions observed during otoscopy. Finally, we enrolled 809 workers in the current study and conducted the ISI, audiometric, clinical, and tests. We provided each subject for written informed consent for participation. Moreover, physicians who conducted clinical examination explained the method and purpose of this study to the participants. After obtaining informed consent from each subject, tests were performed.

### Data collection

The participants were interviewed by trained physicians using a well-established questionnaire. We examined their age, working period, use of hearing protection devices, snoring, smoking, drinking, physical exercise, medical history, current medical problems, and drug administrations. Each subject was questioned about the use of hearing protection devices. Based on all the responses, we divided the subjects into “No or seldom” and “Always” groups. With respect to snoring, the subjects were asked, “Do you snore when you are sleeping or have you heard from family or friends that you snore when you are sleeping?”. The subjects who answered “Yes” were classified into snoring group, while the rest were classified into non-snoring group. With respect to the smoking status, subjects who were smoking currently were defined as current smokers, and those who had stopped smoking were defined as ex-smoker; the subjects who had never smoked were regarded as non-smokers. Further, subjects who consumed alcohol once or more per week were assigned to the drinking group. Subjects who exercised for 30 min or longer at least three times a week were classified into the physical activity group.

The height, weight, waist circumference, and blood pressure were measured. Height and weight were measured to the nearest 0.1 cm and 0.1 kg, respectively. Waist circumference was measured at the mid-point between the twelfth rib and anterior superior iliac spine. The body mass index (BMI) was calculated by dividing the weight (kg) by the squared height (meter). Blood pressure was measured in the right arm in the sitting position by using digital blood pressure monitor under stable conditions.

Blood samples (drawn from a peripheral vein) of all the participants were collected after 12-h fasting. Total cholesterol, high-density lipoprotein (HDL) cholesterol, triglyceride, and fasting glucose levels were measured. Hypertension was defined as systolic blood pressure ≥ 140 mmHg or diastolic blood pressure ≥ 90 mmHg or antihypertensive medication. Diabetes mellitus was defined as fasting glucose ≥126 g/dL or medication for diabetes.

We reviewed the data of working environment noise measured in 2014, which were provided by the company, and the mean noise level of each department to which the workers belonged was regarded as the noise level of the worker.

### Insomnia assessment

For evaluating insomnia, we conducted the ISI test, which is regarded as a validating measurement worldwide [21, 22]. The Korean version of ISI has also proven to be a reliable and appropriate assessment of the severity of insomnia in Korean populations [23]. The ISI test helps to assess the severity, impact, and nature of insomnia. It consists of seven questions described below

(1) Difficulty in falling asleep; (2) Difficulty staying asleep; (3) Problems on waking up too early; (4) How satisfied/dissatisfied are you with your current sleep pattern?; (5) How noticeable to others do you think your sleep problem is in terms of impairing the quality of your life?; (6) How worried/distressed are you about your current sleep problem?; (7) To what extent do you consider your sleep problem to interfere with your daily functioning currently?

All the participants answered these questions in the form of ratings from 0 to 4 (for example, 0 = no problem, 4 = very severe problem, etc.). We added the scores for all seven questions and obtained a total score ranging from 0 to 28. The total score is interpreted as follows: absence of insomnia (0–7), sub-threshold insomnia (8–14), moderate insomnia (15–21), and severe insomnia (22–28) [21]. We evaluated the total score as a continuous variable. Further, the total score of ISI was dichotomized and was evaluated as a categorical variable as follows: absence and presence of insomnia (0–14 and 15–28, respectively).

### Audiometric tests

Audiometry was performed for measuring the hearing thresholds of participants. Air conduction pure tone thresholds for the frequencies 1–4 kHz were obtained bilaterally by using an Itera II audiometer with TDH-39P headphones by experienced testers. An audiometric device was calibrated according to the guidelines for audiometry of the Korean Occupational Safety and Health Agency [24]. We considered workers to experience hearing impairment when the hearing threshold was greater than 25 dB hearing level in either ear at each frequency that we measured; this was consistent with a study about risk factors for hearing loss in United States adults based on the US National Health and Nutrition Examination Survey [25].

### Statistical analyses

Age, working period, noise level, waist circumference, BMI, blood pressure, total cholesterol, triglyceride, HDL cholesterol, glucose, smoking, alcohol consumption, exercise, hypertension, diabetes mellitus, snoring, use of protection devices, and pure tone audiometry results were compared for those with and without insomnia. The Student t-test for continuous variables and chi-square test for categorical variables were performed. We compared hearing thresholds according to insomnia by using analysis of covariance (ANCOVA) with adjustment for covariates including age, working period, noise level, snoring, use of protection devices, hypertension, diabetes mellitus, smoking, alcohol consumption, regular exercise, waist circumference, and total cholesterol, triglyceride, HDL cholesterol levels.

Multiple logistic regression was applied to examine the relationship between insomnia and hearing impairments with four different models. The models were as follows: (1) adjusted for age; (2) adjusted for age, working period, noise level, use of protection devices, and snoring; (3) adjusted for age, working period, noise level, use of protection devices, snoring, diabetes mellitus, and hypertension; (4) adjusted for age, working period, noise level, snoring, use of protection devices, diabetes mellitus, hypertension, smoking, alcohol consumption, regular exercise, waist circumference, total cholesterol, triglyceride, and HDL cholesterol.

Additionally, we performed multiple logistic regression analysis to evaluate the relationship between the ISI test score and hearing impairments with same models as mentioned above. The ISI test score was analyzed as a continuous variable. Continuous variables were presented as means ± standard deviation (SD), and categorical variables were presented as numbers and percentages. Results were shown as odds ratio (OR) with 95% confidence intervals (CI). Statistical significance was set at *p*-value < 0.05. The SPSS version 23.0 software was utilized for all statistical tests (SPSS Inc., Chicago, IL, USA).

## Results

All the 809 subjects that participated in this study were men. The number of subjects those who were classified as normal hearing were 277 and as hearing impairment were 532. General characteristics of the subjects were presented by hearing status. Age, working period, noise level, snoring, protection device use, ISI score and insomnia were statistically different between two groups. No statistically significant differences were observed between two groups in waist circumference, BMI, blood pressure, total cholesterol levels, triglyceride levels, HDL cholesterol levels, glucose levels, smoking, alcohol consumption, regular exercise, hypertension and diabetes mellitus (Table 1).

**Table 1:** General characteristics of the subjects

Variables	Frequency (%) or mean ± SD		*p*-value^*^
	Normal hearing (*N* = 277)	Hearing impairment (*N* = 532)	
Age (years)	43.7 ± 5.8	46.8 ± 6.0	<0.001
Working period (years)	17.5 ± 6.5	22.8 ± 6.1	<0.001
Noise level (decibel)	79.1 ± 3.6	80.3 ± 2.8	0.010
Waist circumference (cm)	84.8 ± 7.2	85.5 ± 7.2	0.181
Body mass index (kg/m^2^)	24.5 ± 3.0	24.7 ± 2.7	0.304
Systolic blood pressure (mmHg)	125.7 ± 11.1	125.5 ± 9.3	0.781
Diastolic blood pressure (mmHg)	81.8 ± 6.9	81.7 ± 6.9	0.809
Total cholesterol (mg/dL)	195.1 ± 32.5	196.1 ± 34.2	0.677
Triglyceride (mg/dL)	167.6 ± 127.4	162.3 ± 108.4	0.533
HDL cholesterol (mg/dL)	47.1 ± 9.9	48.0 ± 11.0	0.295
Glucose (g/dL)	105.3 ± 17.3	108.4 ± 24.4	0.063
ISI score	7.9 ± 4.8	8.7 ± 5.3	0.039
Smoking			0.863
Non-smoker	61 (22.0)	120 (22.6)	
Ex- or current smoker	216 (78.0)	412 (77.4)	
Drinking			0.924
Non-drinker	71 (25.6)	138 (25.9)	
Drinker	206 (74.4)	394 (74.1)	
Exercise			0.892
Physical inactivity	94 (33.9)	178 (33.5)	
Physical activity	183 (66.1)	354 (66.5)	
Hypertension			0.058
No	262 (94.6)	483 (90.8)	
Yes	15 (5.4)	49 (9.2)	
Diabetes mellitus			0.329
No	268 (96.8)	507 (95.3)	
Yes	9 (3.2)	25 (4.7)	
Snoring			<0.001
No	208 (75.1)	280 (52.6)	
Yes	69 (24.9)	252 (47.4)	
Protection device use			0.001
No or seldom	98 (35.4)	255 (47.9)	
Always	179 (64.6)	277 (52.1)	
Insomnia			0.022
No	255 (92.1)	461 (86.7)	
Yes	22 (7.9)	71 (13.3)	

^*^ *p*-value was calculated by Student t-test and chi-square test

**Fig. 1:**
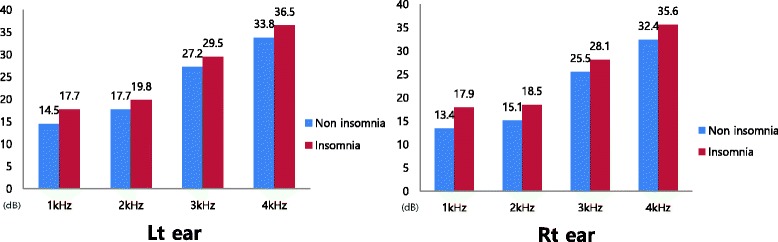
Hearing thresholds of the subjects according to insomnia. * Bar: the mean of hearing threshold of the both ear at all frequency

Based on insomnia, pure tone audiometry thresholds at 1–4 kHz at both ears were presented. The pure tone audiometry thresholds at all frequencies for both the ears were higher for the non-insomnia group compared to the insomnia group. No significant correlation was observed between the hearing thresholds for the left ear, 3 and 4 kHz in the right ear, and insomnia status. However, at 1–2 kHz in the right ear, the hearing thresholds were significantly high for the insomnia group (Fig. 1).

We analyzed the data using ANCOVA after adjusting the covariates such age, working period, noise level, snoring, use of protection devices, hypertension, diabetes mellitus, smoking, alcohol consumption, regular exercise, waist circumference, total cholesterol, triglyceride, and HDL cholesterol. Pure tone audiometry thresholds at 1–2 kHz in the right ear and at 1 kHz in the left ear were significantly high among workers with insomnia (Table 2).

**Table 2:** Comparisons of pure tone audiometry threshold according to insomnia

Frequency	Mean ± SD		*p*-value^*^	*p*-value^†^
	Non insomnia (*N* = 716)	Insomnia (*N* = 93)		
Left ear				
1 kHz	14.5 ± 9.8	17.7 ± 12.6	0.068	0.026
2 kHz	17.7 ± 12.6	19.8 ± 14.9	0.139	0.191
3 kHz	27.2 ± 19.2	29.5 ± 19.3	0.271	0.342
4 kHz	33.8 ± 21.2	36.5 ± 20.4	0.242	0.351
Right ear				
1 kHz	13.4 ± 9.0	17.9 ± 15.4	0.007	0.001
2 kHz	15.1 ± 10.8	18.5 ± 14.2	0.028	0.004
3 kHz	25.5 ± 17.9	28.1 ± 18.5	0.190	0.256
4 kHz	32.4 ± 20.9	35.6 ± 22.0	0.174	0.255

^*^ *p*-value was calculated by Student t-test

^†^ *p*-value was calculated by analysis of covariance and adjusted for age, working period, noise level, snoring, protection device use, hypertension, diabetes mellitus, smoking, alcohol consumption, regular exercise, total cholesterol, triglyceride, HDL cholesterol and waist circumference

Multiple logistic regression analysis of the ISI test score for hearing impairments was performed. The ISI test score increased by 1 point, and the OR for hearing impairments was 1.032 (95% CI: 1.001–1.064) after adjustment for age. However, in three different models, the OR of the ISI test scores for hearing impairments were 1.026 (95% CI: 0.994–1.060), 1.026 (95% CI: 0.994–1.060), and 1.025 (95% CI: 0.992–1.059), respectively, which were statistically insignificant (Table 3).

**Table 3:** Odds ratio of Insomnia Severity Index score^a^ for hearing impairments in separate adjusted models

	B (S.E.)	OR	95% CI	*p*-value
Model 1	0.031 (0.016)	1.032	1.001 to 1.064	0.045
Model 2	0.026 (0.016)	1.026	0.994 to 1.060	0.112
Model 3	0.026 (0.016)	1.026	0.994 to 1.060	0.116
Model 4	0.025 (0.017)	1.025	0.992 to 1.059	0.135

^a^The unit of Insomnia Severity Index score is 1 point

Model 1; adjusted for age

Model 2; adjusted for age, working period, noise level, protection device use and snoring. Model 3; adjusted for age, working period, noise level, protection device use, snoring, diabetes mellitus and hypertension

Model 4; adjusted for age, working period, noise level, snoring, protection device use, diabetes mellitus, hypertension, smoking, alcohol consumption, regular exercise, total cholesterol, triglyceride, HDL cholesterol and waist circumference

Multiple logistic regression of insomnia for hearing impairments was analyzed in an age-adjusted model, and it was found that insomnia was associated with 2.033-fold increase in the odds of hearing impairment (95% CI: 1.190–3.476). Additional adjustment for working period, noise level, use of protection devices, and snoring was performed, and the OR of insomnia for hearing impairments was found as 1.834 (95% CI: 1.043–3.225). After additionally adjusting for diabetes mellitus and hypertension, the OR of insomnia for hearing impairments changed to 1.842 (95% CI: 1.046–3.242).

Finally, adjustment for age, working period, noise level, snoring, use of protection devices, diabetes mellitus, hypertension, smoking, alcohol consumption, regular exercise, waist circumference, and total levels of cholesterol, triglyceride, HDL cholesterol revealed that the OR of insomnia for hearing impairments was 1.806 (95% CI: 1.022–3.188) (Table 4).

**Table 4:** Odds ratio of insomnia^a^ for hearing impairments in separate adjusted models

	B (S.E.)	OR	95% CI	*p*-value
Model 1	0.710 (0.274)	2.033	1.190 to 3.476	0.009
Model 2	0.606 (0.288)	1.834	1.043 to 3.225	0.035
Model 3	0.611 (0.289)	1.842	1.046 to 3.242	0.034
Model 4	0.591 (0.290)	1.806	1.022 to 3.188	0.042

^a^Insomnia group was defined as more than Insomnia Severity Index score 14 points

Model 1; adjusted for age

Model 2; adjusted for age, working period, noise level, protection device use and snoring. Model 3; adjusted for age, working period, noise level, protection device use, snoring, diabetes mellitus and hypertension

Model 4; adjusted for age, working period, noise level, snoring, protection device use, diabetes mellitus, hypertension, smoking, alcohol consumption, regular exercise, total cholesterol, triglyceride, HDL cholesterol and waist circumference

## Discussion

In this study, we focused on the influence of insomnia on hearing impairment and showed that the presence of insomnia (as evaluated by the ISI test) elevated the risk of hearing impairment after adjustment for covariates (OR 1.806; 95% CI: 1.022–3.188). Further, pure tone audiometry thresholds at low frequency levels (1 kHz in the left ear and 1–2 kHz in the right ear) were significantly high in workers with insomnia.

No previous studies have been conducted to examine the association of insomnia and hearing loss owing to the exposure to noise. Only few studies have suggested the relationship between sleep and hearing problems including hearing loss and hearing complaints. A large-scale study with 9756 working individuals in Sweden indicated statistically significant differences in the prevalence of hearing problems among individuals with different levels of sleep quality, and poor sleep quality was found to be associated with hearing problems in both men and women. However, this study used questionnaire-based subjective hearing complaints as an outcome variable, and the actual hearing loss was not evaluated [18]. Another cross-sectional study with 298 male workers occupationally exposed to harmful noise in Israel showed connection between sleep quality and hearing impairment due to prolonged exposure to industrial noise; however, this study highlighted influence of hearing impairment on sleep quality [19]. A Korean study conducted on 198 workers exposed to aircraft noise showed no significant association between sleep quality and NIHL; however, the researchers had evaluated hearing threshold at only 4 kHz [20]. Therefore, former studies about association between sleep quality and hearing loss showed inconsistent results, the correlation between the two is not clear so far.

The nature of the association between insomnia and hearing impairment is unclear, and the underlying mechanism of insomnia in the pathogenesis of hearing impairment is not elucidated. A plausible mechanism linking insomnia and hearing impairment is oxidative stress. An experimental study by Yamane H showed that superoxide levels increased along with the luminal membrane of the marginal cells of the stria vascularis after an exposure to high-intensity rock and roll music [26]. Further, numerous studies have revealed that reactive oxygen species (ROS) and free radicals can trigger hair cell death by damaging DNA and breaking down lipids and proteins [27–29]. Moreover, a study by Bielefeld suggested that increased superoxide levels lead to hair cell death, and this is a sufficient cause of hearing impairment [30]. In similar previous studies, the association of increased ROS levels and hearing impairment has been proven. A case-control study performed by Gulec investigated the effects of primary insomnia on increment of oxidative stress by measuring oxidative stress biomarkers and showed that the patients with primary insomnia had low glutathione peroxidase activity and higher malondialdehyde levels compared with the healthy volunteers [31]. Therefore, there is a possibility that increased oxidative stress by insomnia can intensify hearing impairment of workers exposed to harmful noise.

Another mechanism that might explain the association of insomnia and hearing impairment is the change in the sympathetic tone. Insomnia, shortness of sleep, and sleep deprivation increase the sympathetic activity and can lead to alteration of blood flow [32, 33]. The cochlea is anatomically vulnerable to ischemia as the blood flow is supplied by an end artery [34, 35]. Thus, alteration of the sympathetic tone might lead to the decrement of blood supply to cochlea and cause hearing impairment.

In our study, although pure tone audiometry thresholds at 1–4 kHz were high among workers with insomnia, only thresholds at 1–2 kHz in the right ear and at 1 kHz in the left ear were statistically significant. The arteries, which feed the stria vascularis located in the lateral wall of the cochlea are few in number at the apex than at the base. Thus, the hair cells at the apex, which react to the relatively low frequency sounds, could be more vulnerable to ischemia [34].

Many studies suggest that shift work and sleep deprivation or insomnia are relevant [36, 37]. All subjects in this study were working in the same type of shift. Thus, it is assumed that the ISI score is relatively high for the shift workers, but they were not actually tested. In order to investigate the degree of insomnia induced by shift work and the relationship of hearing loss, it is necessary to include daytime employees in later studies.

The strength of our study was the homogeneity of the participants who worked in the same workplace; this allowed comparisons of hearing impairment and insomnia under similar circumstances. Another strength was that all 809 participants were tested by the same method and equipment. Adjustment of noise-exposure level is very important for evaluation of hearing loss since noise is one of the most significant risk factors for the development of hearing loss. In this study, we utilized the objective noise exposure measurement data that were provided by each department; this could possibly be another strength of our study.

Our study has several limitations. First, owing to the cross-sectional design, we could not confirm whether insomnia precedes and leads hearing impairment; hence, we could not clarify the exact order of the relationship. Possibly, insomnia is triggered by hearing impairments and tinnitus. Second, we attempted to control several confounding factors, which could affect hearing loss; however, not all possible factors could be controlled, which included the environmental noise near the residence of participants. Third, since all the workers in the tire-manufacturing factory were men, our study included all male participants. Sex is one of the most clearly identified demographic risk factors of insomnia. Moreover, insomnia is more prevalent in women than in men [38]. Further research is required to assess the impact of insomnia on hearing impairment with respect to sex. Fourth, insomnia was evaluated only once at the beginning of the study and we did not follow up. For that reason, we could not consider the probable effects of change in the severity of insomnia. Fifth, Investigations about the tinnitus of the subjects were not conducted carefully. The relationship between tinnitus and hearing loss has already been studied in a number of studies, but this study did not provide an accurate information on tinnitus, and it was difficult to elucidate the relationship between insomnia, tinnitus, and hearing loss. This point should be considered in future studies.

The present study is the first to examine the association between insomnia and hearing impairment in workers exposed to occupational noise. Although there are some limitations as mentioned above, we have attempted to demonstrate the relationship between insomnia and degree of hearing impairment. Further long-term prospective studies are necessary to supplement the limitations of this study.

## Conclusions

In conclusion, our study showed that insomnia could be associated with hearing impairment and might affect the degree of hearing loss in workers who are exposed to occupational noise. Additionally, it was shown that insomnia may be associated with decreased hearing at lower frequencies. Especially, more efforts are required to improve the quality of sleep for workers who are exposed to loud occupational noise. However, the precise mechanisms of effects of insomnia on hearing impairment have not been established. In the future, additional prospective investigation is required to identify the mechanism of the development of hearing impairment in workers with insomnia.

## Abbreviations

ANCOVA: Analysis of covariance
BMI: Body Mass Index
CI: Confidence interval
HDL: High-density lipoprotein
ISI: Insomnia Severity Index
NIHL: Noise-induced hearing loss
OR: Odds ratio
